# Fyn Signaling in Ischemia-Reperfusion Injury: Potential and Therapeutic Implications

**DOI:** 10.1155/2022/9112127

**Published:** 2022-09-15

**Authors:** Fang Du, Tao Tang, Qingzhu Li, Jiaxin Liu

**Affiliations:** ^1^Medical School of Kunming University of Science and Technology, Kunming, Yunnan 650500, China; ^2^Yan'an Hospital of Kunming, Kunming Yunnan 650500, China

## Abstract

Ischemic stroke caused by arterial occlusion is the most common type of stroke and is one of the leading causes of disability and death, with the incidence increasing each year. Fyn is a nonreceptor tyrosine kinase belonging to the Src family of kinases (SFKs), which is related to many normal and pathological processes of the nervous system, including neurodevelopment and disease progression. In recent years, more and more evidence suggests that Fyn may be closely related to cerebral ischemia-reperfusion, including energy metabolism disorders, excitatory neurotoxicity, intracellular calcium homeostasis, free radical production, and the activation of apoptotic genes. This paper reviews the role of Fyn in the pathological process of cerebral ischemia-reperfusion, including neuroexcitotoxicity and neuroinflammation, to explore how Fyn affects specific signal cascades and leads to cerebral ischemia-reperfusion injury. In addition, Fyn also promotes the production of superoxide and endogenous NO, so as to quickly react to produce peroxynitrite, which may also mediate cerebral ischemia-reperfusion injury, which is discussed in this paper. Finally, we revealed the treatment methods related to Fyn inhibitors and discussed its potential as a clinical treatment for ischemic stroke.

## 1. Introduction

Stroke is an acute cerebrovascular disease with limited brain tissue necrosis or encephalomalacia caused by blood supply disorders in the brain (stenosis or occlusion of blood supply arteries and inadequate blood supply to the brain) [1]. According to the statistics, there are 2.4 million new stroke patients in my country every year. With the aging of the population, the incidence rate is increasing year by year, and it is the first fatal disease of the Chinese people [2, 3]. It includes both ischemic and hemorrhagic strokes, and the majority of which are ischemic. Ischemic strokes account for 60% to 70% of all strokes. Ischemic stroke is the leading cause of disability and death [4]. It is characterized by high morbidity, high disability, and high recurrence rates. Ischemic stroke is caused by interruption of cerebral blood flow or blockage of cerebral blood vessels by a thrombus, resulting in local cerebral hypoxia and glucose deficiency, which can eventually lead to devastating and irreversible brain damage [5].

**Figure 1:**
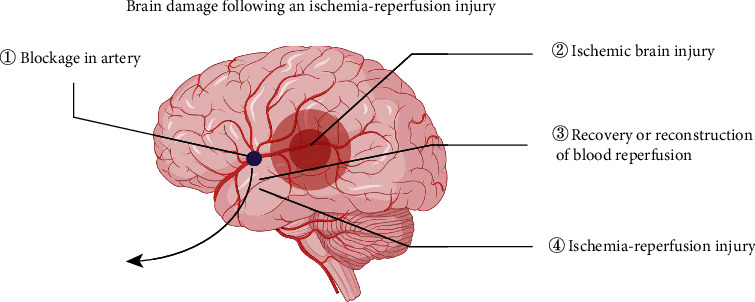
Ischemia-reperfusion injury. Ischemic injury due to blocked blood vessels and reperfusion injury when blood flow is restored.

Early recovery or reconstruction of blood reperfusion is the main treatment plan for ischemic stroke, which is also called cerebral ischemia-reperfusion (I/R) [6]. Treatment for ischemic stroke focuses on recanalization of the occluded blood vessel as soon as possible to limit brain injuries and salvage threatened brain tissue. However, reperfusion injury persists in many patients despite the rapid restoration of blood vessel patency, which has led to the concept of reperfusion injury [7]. This phenomenon of aggravated brain tissue damage caused by the restoration of blood perfusion is called cerebral ischemia-reperfusion injury (CIRI) [8, 9]. CIRI consists of complex mechanisms and a cascade network, ranging from the ischemic time duration to reperfusion. The physiopathological of CIRI is known to involve neuroexcitotoxicity, neuroinflammation, and neuronal cell damage is inevitable consequences of focal cerebral ischemia in the area of cerebral infarction [10]. There is evidence linking oxidative stress, neural excitotoxicity, calcium overload, and inflammation not only accompany the pathological development of I/R but are also major causes of neuronal death [11, 12]. The damage of ischemia-reperfusion is shown in Figure 1.

CIRI is a highly complex cascade reaction process. Reconstruction of blood flow in ischemia-stricken tissue enhances neuroexcitotoxicity, and oxidative stress generates large amounts of ROS and leads to an increased release of inflammatory factors, triggering a series of pathological cascades [13, 14].

At present, the pathological mechanism of CIRI has not been fully elucidated. Therefore, researching and revealing the key molecules of CIRI is of great significance for patients' early prevention and treatment [15]. The tyrosine kinase Fyn may be one such promising target due to its diverse role in the human nervous system.

The tyrosine kinase Fyn is a member of the Src family kinases (SFK), which is widely expressed in the brain tissue and is involved in the regulation of corticogenesis, oligodendrocyte maturation, myelination, neural cell migration, cytokine production, long-term potentiation, excitatory, and inhibitory neuronal receptors. [16, 17]. Previous studies have confirmed that Fyn can regulate inflammation, apoptosis, pyroptosis, oxidative stress, and mitochondrial function through various signaling pathways [18, 19]. Similar to other SFK proteins, Fyn has two key tyrosine residues located, Y531 and Y420. Y420 is located in the activation loop of the kinase domain, and when this site is phosphorylated, Fyn is activated to interact with other proteins [16]. Phosphorylation and dephosphorylation of Y420 and Y531 regulate the activity of Fyn and its ability to interact with other proteins [20, 21].

In adult rodents, Fyn has been demonstrated to be involved in ischemic brain injury. Src or Fyn knockdown decreased neuronal cell passing in a glucose-oxygen hardship cell model [22]. With the top-to-bottom investigation of Fyn in the field of neurological capacity, Fyn has been affirmed to assume a critical administrative part in neurodevelopment and neuroinflammation. In grown-up rodents, Src or Fyn knockdown can reduce hypoxia-induced neuronal cell death and have neuroprotective effects [23]. Fyn can likewise be associated with TLR4 to advance cerebral ischemia-incited neuroinflammation [24]. Fyn intercedes excitotoxicity, neuroinflammation, and the creation of ROS by enacting numerous flagging pathways, in this way irritating ischemia-reperfusion injury.

Studies have shown that Fyn interacts with N-methyl-D-aspartate receptor (NMDAR), postsynaptic density protein 95 (PSD95), L-type voltage-gated calcium channel (LVGCC), and GTPase activation at synapses protein (synaptically localized GTPase-activating protein, SynGAP) interact with each other to further exacerbate brain injury [25–27]. NMDA receptors are heteromultimers composed primarily of NR1 and NR2 (NR2A-NR2D) subunits. Fyn has been identified in the NMDAR complex and has been shown to promote tyrosine phosphorylation of the NR2A and NR2B [28]. PSD-95 functions as a scaffolding protein, clustering NMDARs with other proteins at postsynaptic sites such as neuronal nitric oxide synthetase and SynGAP [29].

Fyn phosphorylates NMDAR and is involved in the regulation of neuronal growth and function, including the induction of ischemic brain injury. [30, 31]. To exacerbate excitotoxicity, Fyn can also activate the type I IP3 receptor and phospholipase C (PLC) to regulate IP3 production and promote IP3-mediated calcium release [32]. Fyn-mediated NMDAR tyrosine phosphorylation is also involved in the regulation of the susceptibility of kindling and seizure. Calcium influx induced by NMDAR activation can regulate NADPH oxidase to generate superoxide through downstream signaling pathways, which combines with nitric oxide (NO) to form highly cytotoxic peroxynitrite and receptive oxygen species (ROS) [33, 34].

Activated Fyn can phosphorylate PKC*δ*, and the Fyn-PKC signaling axis can further activate MAP kinase phosphorylation and NF-*κ*B pathway, indicating that Fyn is a proinflammatory major upstream regulator of signaling [35].

Activated Fyn can also phosphorylate the nonreceptor tyrosine kinase Proline-rich tyrosine kinase 2 (proline-rich tyrosine kinase 2, Pyk2, also known as PTK2B and FAK2) [36]. It has a certain relationship with the change of intracellular calcium ion concentration and can be activated through a calcium-dependent mechanism and participate in the downstream signal transmission of Ca^2+^ [37]. Phosphorylation of Pyk2 is increased in ischemic brain injury, and phosphorylation of Pyk2 promotes brain injury-induced neuroinflammation [38].

This review will discuss the effects of Fyn and its downstream signaling pathways on ischemia-reperfusion injury and its related mechanisms and provide therapeutic strategies for alleviating ischemia-reperfusion injury and improving the prognosis of ischemic stroke.

## 2. Neuroexcitotoxicity and Fyn

During ischemia, glutamate reuptake mechanisms are impaired due to energy deprivation, and as a result, postsynaptic glutamate receptors are chronically hyperactivated. Excitotoxicity is a deleterious cellular process caused by intracellular calcium (iCa^2+^) overload and subsequent dysregulation of the N-methyl-D-aspartate receptor (NMDAR) [39]. NMDARs are highly permeable to Ca^2+^, which is essential for excitatory neurotransmission and synaptic plasticity in the nervous system. NMDARs are mobile within the plasma membrane and can diffuse laterally between synaptic and extrasynaptic sites. Under pathological conditions, alterations in the location and function of NMDARs and disruption of the balance between downstream kinases and phosphatases may occur, leading to neuronal death [40]. Excitotoxic mediated by NMDARs is a key mechanism in ischemia-induced damage [41]. Excessive release of neurotransmitters mediated by calcium ion influx causes excitotoxic injury [33].

The main pathways of calcium influx are voltage-sensitive calcium channels (VSCC) and N-methyl-D-aspartate linked receptor-operated calcium channels (N-methyl-D-aspartate linked receptor-operated) [42]. There are four types of VSCC in neurons, namely T, L, N, and P types [43]. During ischemia, the lack of ATP in neurons will inhibit the Na^+^-K^+^ pump, resulting in the accumulation of Na^+^, promoting the exchange of Na^+^-Ca^2+^, and increasing the concentration of intracellular Ca^2+^ [42]. Overstimulation of NMDA receptors by glutamate during ischemia is the main pathway for the lethal influx of Ca^2+^, and VSCC is also activated during ischemia, which also causes Ca^2+^ influx [44].

When Ca^2+^ influx through NMDARs leads to the production of superoxide and nitric oxide, which react to form highly cytotoxic peroxynitrite. In the brain, nitric oxide is produced by neuronal (nNOS) nitric oxide synthase [33]. On the other hand, recent studies have shown that NMDAR activation-induced superoxide originates primarily from NADPH oxidase (NOX). In neurons, the main NOX isoform is NOX2. The coupling between NMDAR activation and NOX2 activation is mainly through the activation of inositol phosphate 3-kinase (PI3K). PI3K activates neuronal NOX2 by activating PKC*ζ* to generate superoxide. Activation of PI3K leads to phosphatidylinositol (4,5)-biphosphate to generate the second messenger phosphatidylinositol (3,4,5)-triphosphate (PIP3), which can activate atypical protein kinase C (PKC*ζ*) [45].

Phosphorylation of NMDARs and type I IP3 receptors (IP3R1) is regulated by Fyn. Fyn can positively regulate 1,4,5-triphosphate (IP3)-mediated calcium release by phosphorylating IP3R1. Activation of phospholipase C*γ* (phospholipase C*γ*, PLC*γ*) to generate IP3. Selective knockout of Fyn or use of Src inhibitors also attenuates IP3-mediated calcium release and induces autophagy [46].

Fyn phosphorylates NR2B at Tyr531 and Tyr1472. When Fyn is phosphorylated at Tyr531, it can inhibit the phosphorylation of NR2B at Tyr1472, thereby inhibiting the extracellular effect of NR2B [47]. Phosphorylation of Y1472 NR2B by Fyn mediates cell death by increasing reactive oxygen species (ROS) production. Activated Fyn phosphorylates NMDAR and mediates the interaction between NMDAR and PSD-95, which is required for induced neural excitotoxicity. Fyn also promotes the phosphorylation of NR2B at Y1472, preventing AP-2 binding. Removal of the receptor from the synapse requires activator protein-2 (AP-2) binding, thereby altering the internalization of NR2B at the postsynaptic membrane [48].

Overactivation of NMDARs triggers calpain hyperactivation, which in turn leads to TrkB-FL truncation, dysregulated BDNF/TrkB signaling, loss of dendritic spines, and apoptosis [49]. Ischemia-reperfusion injury can release IL-6 into cerebrospinal fluid by activating NMDARs and upregulating endothelin-1 (ET-1) and JNK [50]. ET-1 is a vasoactive peptide. ET-1 is released from ischemic tissue during reperfusion, which may lead to reperfusion injury in adjacent intact tissue [51]. ET-1 can affect the concentration of intracellular Ca^2+^ by directly stimulating the release of Ca^2+^ from the cell, activating PKC to enhance the Ca^2+^ flow of L-type calcium channels, activating Na + -H+ exchange, thereby inversely activating Na^+^-Ca^2+^ exchange [52]. Na + - Ca^2+^ exchange is the main cause of ischemia/reperfusion injury.

In vivo, endogenous NO synthesized by NO synthases (NOS) can promote the s-nitrosylation of Fyn through s-nitrosoglutathione (GSNO) [53, 54]. Global cerebral ischemia-reperfusion promotes a massive increase in glutamate release and activates glutamate receptors, including NMDAR. In ischemic neurons, glutamate increases intracellular Ca concentration by activating NMDAR, resulting in a sustained increase in nNOS activity. Activation of nNOS can lead to the production of endogenous NO, while the intermediate of NO activates guanylate cyclase. The increase of cyclic guanosine monophosphate (cGMP) and the activation of downstream signaling pathways further aggravate CIRI. [55].

Several studies have found that Fyn can phosphorylate and activate mitochondrial dynamin-related protein 1 (Drp1) through PKC*δ*, thereby regulating apoptosis and inflammatory responses. Fyn stimulates NADPH oxidase through the PKC pathway to increase NADPH oxidase-dependent mitochondrial ROS [56].

Fyn overexpression also accelerates cognitive impairment in AD model mice, and depletion of Fyn or inhibition of Fyn restores memory function and synaptic density in AD model mice [57]. In the cerebral hemorrhage model, cell apoptosis was reduced after downregulation of Fyn; apoptosis-related proteins AIF, Cyt c, caspase 3, and Bax were all downregulated; anti-apoptosis-related protein Bcl-2 was up-regulated, and tunnel staining was reduced [58]. The Drp1 inhibitor Mdivi-1 (Mitochondrial division inhibitor 1) can reverse the proapoptosis induced by Fyn overexpression. Fyn activates Drp1 signaling by phosphorylating 616 serine in Drp1 to increase neuronal apoptosis in rats after intracerebral hemorrhage [59].

## 3. Inflammation and Fyn

Neuroinflammation plays a key role in the pathogenesis of ischemic stroke and other forms of ischemic brain injury that result in neuronal damage and dysfunction [60]. Ischemic stroke triggered inflammatory cascades and further enlarged secondary brain injury due to cytotoxic neuronal cell death and neurological dysfunction. Hence, it is of great significance to elucidate the molecular mechanism of inflammation regulation in CIRI for the treatment and outcome of ischemic stroke [61].

Inflammation accompanies ischemia and reperfusion processes and participates in the pathological process of injury. Ischemia maintains an intravascular inflammatory environment by activating leukocytes and inducing the release of proinflammatory cytokines from vascular endothelial cells [62]. Ischemia also leads to the production of reactive oxygen species (ROS) [63]. Cerebral ischemia leads to disruption of the blood-brain barrier (BBB), which is consistent with increased Fyn activity [64]. The release of proinflammatory factors alters the permeability of the blood-brain barrier (BBB) and leads to the migration of monocytes, neutrophils, and lymphocytes into the brain parenchyma [65]. Fyn may exacerbate cerebral ischemic injury both by activating downstream inflammatory pathways and by causing or contributing to brain edema through BBB disruption [66, 67].

Reoxygenation and glucose replenishment in a tissue previously subject to ischemia boosts oxidative stress and the release of inflammatory mediators and leads to ischemia-reperfusion injury in tissues surrounding the ischemic area. Microglia are resident immune cells in the brain. Microglia can be polarized into different phenotypes after activation. The M1 phenotype has a proinflammatory effect and participates in the occurrence of neuroinflammation, while the M2 phenotype has an anti-inflammatory effect to reduce brain damage caused by inflammation. Ischemia can activate Fyn kinase in microglia to produce reactive oxygen species and proinflammatory factors, which activate and polarize microglia to the M1 type. TNF*α*, interleukin-6 (IL-6), and interleukin-1 (IL-1) are released by M1-type microglia to aggravate tissue damage. Elevation of interleukin-6 (IL-6), an inflammatory marker of stroke, has been reported to be a poor prognostic factor. IL-6 is involved in the regulation of oxidative stress and angiogenesis [68]. IL-6 is involved in the NMDA response and affects neurodevelopment.

A growing body of evidence suggests that postischemic inflammation is important in different stages of cerebral ischemia. Neuroinflammation leads to further neuronal death by increasing the expression of inflammatory factors caused by changes in the brain environment after excitotoxic injury and oxidative stress in CIRI. The use of anti-inflammatory strategies in the treatment of ischemic stroke is appealing because they have a broader therapeutic window than the currently popular reperfusion-based approaches.

Recent studies have suggested that neuroinflammation may be key to the development of progressive stroke after reperfusion [69]. Fyn is involved in the pathological process of various neurodegenerative diseases and is closely related to neuroinflammation [70].

Fyn is upregulated in chronic inflammation, and Fyn knockout mice can largely attenuate neuroinflammatory responses induced by MPTP, LPS, or 6-OHDA [71]. Previous studies have found that Fyn is highly expressed in the hippocampus of ischemia-reperfusion injury, and inhibition or knockdown of Fyn significantly reduces the expression of related inflammatory molecules in cerebral ischemia-reperfusion injury. Functional studies have shown that Fyn is required for proinflammatory responses, including cytokine release and inducible nitric oxide synthase (iNOS) activation [72].

Fyn is shown to mediate the production of proinflammatory mediators in mast cells, macrophages, basophils, and natural killer cells [73]. Fyn was shown to be activated upon engagement of fibrillar amyloid peptides by its receptor CD36, contributing to the activation and migration of primary macrophages and microglia, and to the neurotoxicity of BV2 microglia by prion proteins activated by fragment stimulation, etc. Fyn knockout mice have less adipose tissue inflammation due to T cell and macrophage infiltration and a higher proportion of anti-inflammatory M2 macrophages [74].

Fyn can modulate the transduction of inhibitory or activating signals of immune receptors, and Fyn deficiency has protective effects against arthritis and nephritis in mice. Fyn is involved in the initiation of ITAM (immunoreceptor tyrosine-based activation motif) receptor-mediated signaling, and Fyn is responsible for ITAM phosphorylation after receptor aggregation, leading to Syk by recruiting downstream effectors such as PI3-kinase and phospholipase C-*γ* to initiate further signal propagation [35]. Therefore, the activation of Fyn has important significance in neuroinflammation.

Pyk2 localizes to neuronal postsynaptic sites and is involved in the regulation of synaptic plasticity [75, 76]. Pyk2 is thought to be a target of Fyn-specific regulation. The level of Pyk2 tyrosine phosphorylation was substantially increased in the coexpression system of Pyk2 with Fyn [77]. Another study also found increased Pyk2 activity in mice constitutively overexpressing Fyn (FynCA) [78]. Selective regulation of Pyk2 tyrosine phosphorylation by Fyn in vivo was associated with preferential phosphorylation of Pyk2 by Fyn in vitro. Pyk2 knockout mice do not cause significant developmental impairment but affect the cell migration of macrophages and marginal zone B cells [79]. Pyk2 is required for macrophage polarization and migration to sites of inflammation [80].

Pyk2 regulates ASC, an inflammasome adaptor protein that plays a role in innate immune responses and inflammatory diseases by activating auto-oligomerization of the NLRP3 inflammasome [81]. Pyk2 phosphorylation is closely linked to the occurrence and progression of various neurological diseases. Fyn regulates Pyk2 activity, which can hyperphosphorylate tau protein and increase the risk of Alzheimer's disease. Pyk2 inhibition can reduce neuroinflammation by downregulating the expression of Matrix Metallopeptidase 9 (MMP-9), [82]. Activated Pyk2 phosphorylates MCU and increases mitochondrial calcium uptake, resulting in mitochondrial calcium overload and dysfunction [83].

Previous studies have found that Npas4, an immediate early gene, is enhanced in Pyk2 knockout mice, but how it is regulated and its molecular mechanisms remain unclear [84]. Inhibition of Fyn can reduce the expression of inflammatory factors and upregulate the expression of Npas4, which is regulated by Pyk2.

**Figure 2:**
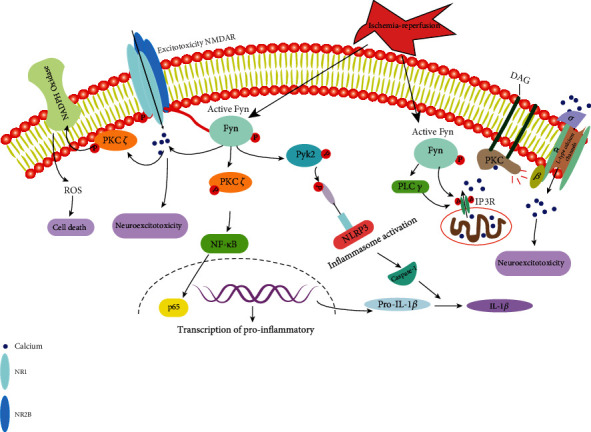
The role of Fyn in reperfusion injury. Increased Fyn activity leads to hyperphosphorylation of the NMDAR2B subunit and IP3R, which leads to increased calcium influx and subsequent excitotoxicity. Fyn phosphorylates PKC*δ*, leading to activation of NF-*κ*B and causing entry of the P65 component into the nucleus. P65 entry into the nucleus leads to transcription of proinflammatory cytokine genes, such as IL-1*β*.

Npas4 may be involved in regulating cell death-associated signaling pathways and inflammatory responses [85]. According to Ooe et al., a knockout mouse model of Npas4 (Npas4-/-) exhibited cumulative neurodegeneration in their brains. An increase in glial fibrillary acidic protein (GFAP) expression indicates the activation of glial cells, which causes nerve damage [86]. The number of activated microglia and astrocytes was significantly increased in Npas4-/-mice 96 h after stroke induction. The study also found that using OGD, knockout of Npas4 in cultured neurons resulted in increased susceptibility to cell death and also found that Npas4-/-mice had significantly larger lesion areas than wild-type mice after induction of cerebral ischemia and neurodegeneration. Npas4 levels were significantly higher, confirming its neuroprotective role in ischemic stroke [87].  Figure 2 presents the mechanism of brain injury induced by Fyn activated by ischemia-reperfusion.

## 4. The Potential of Fyn as a Therapeutic Target for Stroke

Fyn inhibitors have therapeutic effects on many diseases, including tumors, neurological diseases, and osteoarthritis [88–94]. Preclinical studies in rodents with Fyn inhibitors suggest that targeting this kinase family may be beneficial in humans to prevent ischemic brain injury (Table 1 [95].

Saracatinib is an Src inhibitor that also inhibits Fyn, c-Yes, Lyn, BLK, FGR, and Lck and has been shown to reduce glial hyperplasia, neurodegeneration, and nitro-oxidative stress [96]. On123300 is a multi-target kinase inhibitor that inhibits CDK4, ark5/nuak1, PDGFR, FGFR1, RET (c-RET), and Fyn. On123300 has been shown in studies to reduce apoptotic cell death induced by OGD/R, as well as the expression of p-Rb, B-Myb, and Bim and alleviate ischemic/hypoxic nerve injury [97].

PP1 (4-amino-5-(4-methylphenyl)-7-(t-butyl) pyrazolo [3,4-d]pyrimidine) and PP2 (4-amino-5-(4-chlorophenyl)-7-(t-butyl) pyrazolo [3,4-d]pyrimidine), are ATP analogs that compete with ATP for the ATP-binding pocket of SFKs, thereby reducing the ability of SFKs to phosphorylate substrates. Both compounds have certain selectivity for Fyn in SFKs, but PP2 is more selective for Fyn [98].

PP2 protects hippocampal CA1 pyramidal neurons from transient ischemia. Inhibition of Fyn can inhibit calcium overload by promoting the interaction of L-type calcium channels with Bcl-2-related immortal gene 3 (athanogene 3), thereby reducing ischemia-reperfusion injury. Experiments in the adult rat middle cerebral artery occlusion (MCAO) model showed that PP2 reduced infarct volume and Blood-Brain Barrier (BBB) leakage [99]. PP2 blocks ET-1-induced elevation of IL-6 [59]. However, the mechanism of PP2 in alleviating cerebral ischemia-reperfusion injury has not been fully elucidated.

## 5. Conclusion

On the one hand, the upregulation of Fyn through neuromodulation may also benefit several aspects such as neuronal migration, synaptogenesis, and synaptic plasticity [100, 101]. Neuromodulation is essential for synaptogenesis and plasticity [102]. Increasing Fyn activity promotes actin dynamics allowing oligodendrocyte maturation and facilitating synaptic regeneration, and subsequent myelin production [103]. On the other hand, increased levels of Fyn correlate with increased microglia activation in PD brains, and Fyn is also involved in T cell differentiation, particularly through the release of proinflammatory cytokines by Th17 [104]. Considering the damage of inflammatory response on brain function after ischemia-reperfusion, special attention needs to be paid to the changes in Fyn activity due to ischemia-reperfusion.

More and more studies suggest that Fyn plays an important management role in ischemia-reperfusion injury, yet many mechanisms remain unexplained. Fyn inhibitors have been shown to reduce exacerbations. The use of Fyn inhibitors inhibits calcium overload and elevated inflammatory cytokines, thereby reducing ischemic brain injury. Fyn inhibitors have a palliative effect in a variety of diseases involving neuroinflammation. Elucidating the mechanism of Fyn inhibitor is helpful to clarify the molecular mechanism of Fyn affecting the prognosis of ischemia-reperfusion injury and the mechanism of Fyn inhibitor protecting nerve injury from ischemia-reperfusion injury and provides potential therapeutic targets for the treatment of ischemia-reperfusion injury.

## Figures and Tables

**Table 1:** The inhibitors of Fyn and Targets.

Fyn inhibitor	Target	Effects
Saracatinib	c-yes, Fyn, Lyn, Blk, Fgr, and Lck	Alleviate microglia, astrogliosis, neurodegeneration, and nitro-oxidative stress
PP1	Lck/Fyn	Reduced cerebral infarct size and neurologic dysfunction
PP2	Lck/Fyn	Inhibition of BBB leakage and reduction of infarct volume
ON123300	CDK4, Ark5/NUAK1, PDGFR*β*, FGFR1, RET (c-RET), and Fyn	Improve OGD/R induced apoptotic cell death and the expression of p-Rb, B-myb, and Bim

## Data Availability

No original data were used in this study.
